# Visual and Vestibular Inputs Affect Muscle Synergies Responsible for Body Extension and Stabilization in Sit-to-Stand Motion

**DOI:** 10.3389/fnins.2018.01042

**Published:** 2019-01-15

**Authors:** Kazunori Yoshida, Qi An, Arito Yozu, Ryosuke Chiba, Kaoru Takakusaki, Hiroshi Yamakawa, Yusuke Tamura, Atsushi Yamashita, Hajime Asama

**Affiliations:** ^1^Department of Precision Engineering, The University of Tokyo, Tokyo, Japan; ^2^Center of Medical Science, Ibaraki Prefectural University of Health Science, Inashiki, Japan; ^3^Research Center for Brain Function and Medical Engineering, Asahikawa Medical University, Asahikawa, Japan

**Keywords:** sit-to-stand, muscle synergy, sensorimotor, visual, vestibular

## Abstract

The sit-to-stand motion is a common movement in daily life and understanding the mechanism of the sit-to-stand motion is important. Our previous study shows that four muscle synergies can characterize the sit-to-stand motion, and they have specific roles, such as upper body flexion, rising from a chair, body extension, and posture stabilization. The time-varying weight of these synergies are changed to achieve adaptive movement. However, the relationship between sensory input and the activation of the muscle synergies is not completely understood. In this paper, we aim to clarify how vestibular and visual inputs affect the muscle synergy in sit-to-stand motion. To address this, we conducted experiments as follows. Muscle activity, body kinematics, and ground reaction force were measured for the sit-to-stand motion under three different conditions: control, visual-disturbance, and vestibular-disturbance conditions. Under the control condition, the participants stood without any intervention. Under the visual-disturbance condition, the participants wore convex lens glasses and performed the sit-to-stand motion in a dark room. Under the vestibular-disturbance condition, a caloric test was performed. Muscle synergies were calculated for these three conditions using non-negative matrix factorization. We examined whether the same four muscle synergies were employed under each condition, and the changes in the time-varying coefficients were determined. These experiments were conducted on seven healthy, young participants. It was found that four muscle synergies could explain the muscle activity in the sit-to-stand motion under the three conditions. However, there were significant differences in the time-varying weight coefficients. When the visual input was disturbed, a larger amplitude was found for the muscle synergy that activated mostly in the final posture stabilization phase of the sit-to-stand motion. Under vestibular-disturbance condition, a longer activation was observed for the synergies that extended the entire body and led to posture stabilization. The results implied that during human sit-to-stand motion, visual input has less contribution to alter or correct activation of muscle synergies until the last phase. On the other hand, duration of muscle synergies after the buttocks leave are prolonged in order to adapt to the unstable condition in which sense of verticality is decreased under vestibular-disturbance.

## Introduction

Standing from a seated position is a fundamental daily motion. In daily life, people need to change their posture from sitting to standing to perform other activities, such as walking. The population of elderly people is increasing (World Health Organization, 2016), and many elderly people have difficulty performing sit-to-stand motion. Without the ability to perform sit-to-stand motion, human mobility is affected and quality of life decreases. Causes of inhibited movement vary, including decreased muscle force and weakened sensory input. In particular, it is known that visual, vestibular, sensorimotor, and balance function change with age (Lord and Ward, 1994). The focus of this study was human sit-to-stand motion and the analysis of how sensory inputs affect this motion.

Human sit-to-stand motion has been widely studied, and researchers have investigated the relationship between sensory input and sit-to-stand motion. Lord et al. showed that the sit-to-stand movement was affected by sensory information, such as visual input related to the motion speed (Lord et al., 2002). Scholz et al. investigated the relationship between visual or tactile input and the trajectory of the human sit-to-stand motion and revealed that the trajectory was affected by the ability to use sensory input (Scholz et al., 2001). Mourey et al. studied how age and the ability to use visual input affected the motion speed and trajectory of the center of mass during sit-to-stand motion. They revealed that the speed of the center of mass decreased under a visual-disturbance condition, particularly for elderly subjects (Mourey et al., 2000). Regarding the tactile sensation of feet, Cheng et al. showed that the vertical ground reaction force was different between people who succeeded and failed to perform the sit-to-stand motion (Cheng et al., 2014). These studies indicated that human sit-to-stand motion is affected by sensory input.

Numerous studies have been conducted to determine the effect of sensory input on human locomotion and upright postural control. Ivanenko et al. showed that the duration of the several muscle activation in human gait is controlled by tactile sensations in their feet (Ivanenko et al., 2003). Another study showed that visual information is utilized to stabilize posture and avoid obstacles during locomotion (Logan et al., 2010). Fitzpatrick et al. studied human locomotion during vestibular-disturbances and showed that the direction of travel could not be recognized correctly when vestibular input was disturbed (Fitzpatrick et al., 1999). Furthermore, Franz et al. assessed differences in walking strategies between the young and elderly, and they found that the elderly people rely more on visual information to achieve locomotion (Franz et al., 2015). Chiba et al. demonstrated that the standing posture control was affected by visual, vestibular, and tactile sensations (Chiba et al., 2013). Chiba et al. also reviewed the human standing posture control by visual, vestibular, somatosensory, and tactile inputs (Chiba et al., 2016). Kabbaligere et al. studied the effect of visual and vestibular inputs on bipedal posture tasks and they found that sensory reweighting occurred when sensory input was disturbed (Kabbaligere et al., 2017). Claeys et al. investigated how proprioception and visual information change the strategy required to maintain balance (Claeys et al., 2011). In another study, an experiment to evaluate effect of visual, vestibular, and somatosensory contributions to human control of an upright stance (Maurer et al., 2000) was conducted. The results showed that somatosensory input could compensate for other sensory input. These studies showed that sensory input is utilized to achieve motion.

To understand the mechanisms of human movement, the concept of muscle synergy has been widely acknowledged. This is based on the idea that humans do not control all their muscles individually but control sets of muscles, called “muscle synergy” (Bernshtein, 1967). Some research has shown that humans or other species use muscle synergy in various movements (Ting et al., 2015). In most of the previous studies, the muscle activity was measured and the shared muscle synergies from the different movements were extracted. Lemay et al. used muscle stimulation and measured the output force, finding that cats have several sets of synchronized muscle activation (Lemay and Grill, 2004). Torres-Oviedo et al. showed that the muscle synergy of cats was not task-specific but exhibited generality in tasks (Torres-Oviedo et al., 2006). Shared muscle synergy is also found in human manipulation tasks at various speeds (d'Avella et al., 2008) or between affected and unaffected arms of stroke patients (Cheung et al., 2009). Ivanenko et al. showed that a small set of muscle synergies could account for various forms of human locomotion (Ivanenko et al., 2004). Aoi et al. also showed that the timing of muscle synergy activation was adjusted upon contact of a human foot with the ground (Aoi et al., 2010). In terms of the neural evidence of muscle synergy, Takei et al. suggested that muscle synergy was coded in the spinal cord (Takei et al., 2017). Desrochers et al. also validated that muscle synergies for locomotion were primarily controlled by circuits of neurons within the spinal cord (Desrochers et al., 2018). Tresch et al. demonstrated that muscle activity can be divided into muscle synergies using various matrix factorization algorithms (Tresch et al., 2006). These results imply that various movements can be explained using a relatively small number of modules, such as muscle synergies. Our group analyzed muscle activation in human sit-to-stand motion, demonstrated that there are four muscle synergies in the sit-to-stand motion of humans (An et al., 2015), and found that the activation timing of these synergies differs with age (Yang et al., 2017). It was suggested that muscle synergy is controlled by a feedforward signal and sensory feedback (Cheung et al., 2005). Human movement includes feedforward control, which is learned, and feedback control, which fixes the movement based on sensory input (Kandel et al., 2013). Other researchers have investigated the relationship between sensory input and the muscle activity in movements, but the human sit-to-stand motion has rarely been studied from this point of view.

Though, it has been shown that sensory input is utilized to achieve movement, the relationship between sensory input and the sit-to-stand motion has not been completely investigated. Our study was particularly focused on visual and vestibular inputs, and the effect of these sensory inputs on the muscle synergies in the sit-to-stand motion was clarified.

## Materials and Methods

### Muscle Synergy Model

As stated in the Introduction, the muscle synergy hypothesis suggests that humans do not control individual muscles, but they control a synchronized muscle activation, called muscle synergy (Bernshtein, 1967). This indicates that each synergy controls several muscles, and the synergies are controlled by the nervous system. In the synergy hypothesis, two components are defined: spatial and temporal patterns. The spatial pattern indicates the combination of the activated muscles in each synergy, whereas the temporal pattern shows the time-varying weight coefficient of the spatial patterns. The muscle synergy hypothesis suggests that the linear summation of the spatiotemporal patterns generates muscle activity. This can be expressed by the following equations:

$$\begin{array}{l} \;M=WH,\\ M={\left(m_{1},m_{2}\cdots m_{n}\right)}^{\text{T}}=\left(\begin{array}{c} m_{1}\left(t_{0}\right)\cdots m_{1}\left(t_{\text{max}}\right)\\ \vdots \ddots \vdots \\ m_{n}\left(t_{0}\right)\cdots m_{n}\left(t_{\text{max}}\right) \end{array}\right),\\ \;W=\left(\begin{array}{c} w^{1}\cdots w^{k} \end{array}\right)=\left(\begin{array}{c} w_{1}^{1}\cdots w_{1}^{k}\\ \vdots \ddots \vdots \\ w_{n}^{1}\cdots w_{n}^{k} \end{array}\right)\\ H={\left(h^{1}\cdots h^{k}\right)}^{\text{T}}=\left(\begin{array}{c} h^{1}\left(t_{0}\right)\cdots h^{1}\left(t_{\text{max}}\right)\\ \vdots \ddots \vdots \\ h^{k}\left(t_{0}\right)\cdots h^{k}\left(t_{\text{max}}\right) \end{array}\right), \end{array}$$

where **M**, **W**, and **H** denote the muscle activation, spatial pattern, and temporal pattern matrices, respectively. The muscle matrix **M** contains discrete time-varying muscle activation vectors, **m**_*i*_, where *i* (1 ≤ *i* ≤ *n*) denotes the muscle number and *m*_*i*_(*t*) represents muscle activation at time *t* (*t*_0_ ≤ *t* ≤ *t*_max_). Each column of the spatial pattern matrix **W** indicates the individual spatial pattern column **w**^*j*^ of the *j*-th synergy (1 ≤ *j* ≤ *k*). Its elements, $w_{i}^{j}$, represent the relative muscle activation of the *i*-th muscle in the *j*-th synergy. The temporal pattern matrix **H** includes the vectors **h**^*j*^ and their elements *h*^*j*^(*t*) to express the weight of the *j*-th synergy at time *t*. Non-negative matrix factorization (NNMF) was used to obtain spatiotemporal pattern matrices **W** and **H** (Lee and Seung, 1999). In this study, the norm of the vectors **w**^*j*^ is defined as 1.0.

In various previous studies, spatial patterns were assumed to be constant during human movement. This reflects the findings that the spatial patterns are coded in the human spinal cord (Takei et al., 2017; Desrochers et al., 2018) and that the human nervous system integrates sensory information to control the timing and amplitude of these spatial patterns, i.e., the temporal patterns. In our previous study, it was found that there are four spatial patterns in the human sit-to-stand movement, and activation of each pattern is varied temporally depending on the motion strategy. It was hypothesized that sensory information is utilized to control these synergies and achieve control by changing the temporal pattern, i.e., the spatial patterns are the same under all the conditions. We investigated how visual and vestibular information affect muscle synergies.

**Figure 1:**
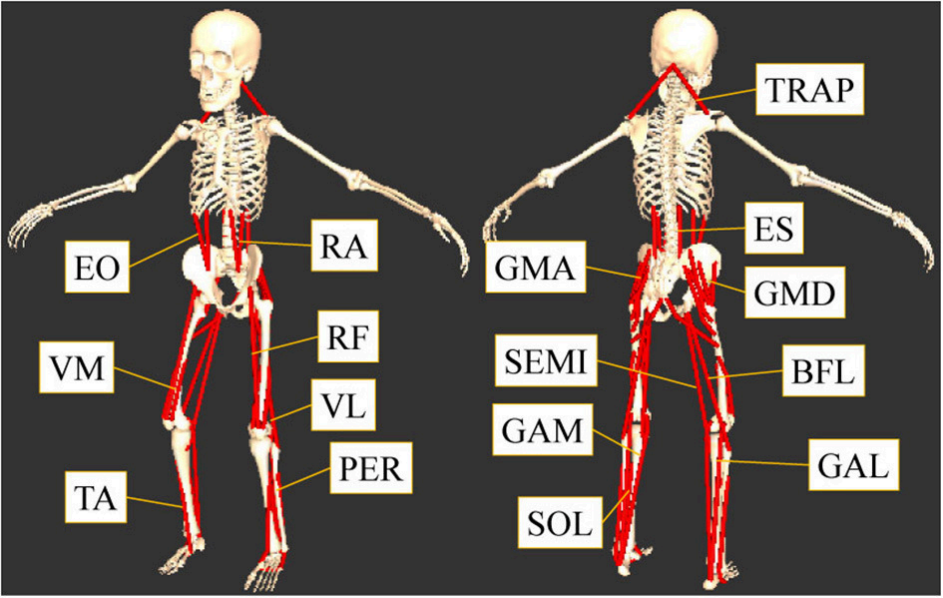
The measured sixteen muscles. This study focused on the muscles that contribute to the flexion or extension of the lumber, hip, knee, and ankle joints: tibialis anterior (TA), gastrocnemius lateralis (GAL), gastrocnemius medialis (GAM), soleus (SOL), peroneus (PER), rectus femoris (RF), vastus lateralis (VL), vastus medialis (VM), biceps femoris long head (BFL), semitendinosus (SEMI), gluteus maximus (GMA), gluteus medius (GMD), rectus abdominis (RA), external oblique (EO), elector spine (ES), and trapezius (TRAP).

This study was focused on the muscles that contribute to the flexion or extension of the lumbar, hip, knee, and ankle joints. The muscles considered are shown in Figure 1. The 16 muscles in the trunk and lower limbs measured from both sides of the body (Figure 1) were: tibialis anterior (TA), gastrocnemius lateralis (GAL), gastrocnemius medialis (GAM), soleus (SOL), peroneus (PER), rectus femoris (RF), vastus lateralis (VL), vastus medialis (VM), biceps femoris long head (BFL), semitendinosus (SEMI), gluteus maximus (GMA), gluteus medius (GMD), rectus abdominis (RA), external oblique (EO), elector spine (ES), and trapezius (TRAP).

### Experiment Procedure

To clarify the effects of visual and vestibular inputs on the muscle synergies in sit-to-stand motion, the muscle synergy structures were compared for a condition with no disturbances (control condition) and conditions with disturbed visual or vestibular inputs.

First, we measured the sit-to-stand motion of the participants under the control condition. No disturbance was added to the visual input, and the subjects were asked to open their eyes and gaze straight forward. Next, the subjects were asked to wear convex lenses (Frenzel glasses) to disturb their visual inputs under the visual-disturbance condition. The convex lenses changed the focus of the eyes, and thus, the participants could not see the surrounding environment. In addition, the light of the room was turned off to inhibit visual input. Under this condition, the participants were asked to open their eyes while standing up from a chair. Having the subjects wear convex lenses rather than simply closing their eyes prevented the weight-shift effect of the sensory information and clarified the difference between the control condition and visual-disturbance condition. A previous study suggested that several sensory inputs are utilized and integrated to recognize the environment and achieve movement (Chiba et al., 2016), and it is known that the weight of a sensory input can be adaptively changed depending on the situation (Nashner and Berthoz, 1978). If the participants closed their eyes voluntarily, this action might induce a weight shift of the sensory information. Therefore, in this study, convex lenses, and a dark room were used to inhibit the visual input to avoid the weight shift for the sensory information.

Finally, we performed caloric tests to disturb the vestibular input (vestibular-disturbance condition) (Baloh and Jen, 2011). Angular acceleration of the head movement are detected by the movement of the lymph in semicircular canals, which are used for detecting and controlling head posture. To disturb this vestibular input, icy water (0°C, 5 mL) was put in the subjects' ears to cause convection of the lymph and stimulate the semicircular canals. When a caloric test successfully modulates the vestibular input, acceleration is unable to be sensed correctly and it is known that nystagmus occurs (Peterka et al., 2004; Indovina et al., 2005; de Lahunta and Glass, 2009). In these experiments, the occurrence of nystagmus was confirmed for all the participants. Under this condition, the participants wore the convex lenses used in the visual-disturbance condition to avoid the usage of visual input. When the surrounding environment is recognized by vision, visual input is used to correct the vestibular-disturbance so that it only has a minor effect (Chiba et al., 2013). Consequently, both visual and vestibular inputs were disturbed. When the vestibular input is disturbed by caloric tests, humans are known to lean to the side where the icy water is inserted. Therefore, the differences between the left and right side of the body were analyzed under the vestibular-disturbance condition.

All the subjects performed sit-to-stand motion under the three conditions in the following order: control, visual-disturbance, and vestibular-disturbance conditions. In all the conditions, subjects were asked to sit on a chair with a height equal to their knee height and were asked to stand up at a comfortable speed. The participants placed their feet in comfortable positions. They had their arms crossed in front of their chest while performing the sit-to-stand motion. Ten trials were performed under the control and visual-disturbance conditions. Under the vestibular-disturbance condition, 2–3 trials were conducted for each participant. The number of trials differed for the participants because some of them could not finish all the trials due to dizziness.

### Data Recording and Signal Processing

The muscle activity, kinematics, and reaction force were measured using a surface electromyography (EMG) sensor (MiniWaveInfinity, Cometa srl.), an optical motion capture (MAC3D, MotionAnalysis Corp.), and force plates (TF4060, TechGihan Corp.), respectively. The surface EMG was recorded at 2,000 Hz, and the data was filtered through a band-pass filter (fourth-order Butterworth filter) from 20 to 500 Hz. Subsequently, a second-order Butterworth filter with a cut-off frequency of 5.3 Hz was used to filter the surface EMG data. Individual muscle activation was normalized to 1.0 based on the maximum activation of all the trials under all the three conditions for each subject. Reflective markers were attached to the participants' bodies based on the Helen Hayes marker set. The recorded marker position data was filtered with a second-order Butterworth low-pass filter with a cut-off frequency of 6 Hz to remove the noise. Using the musculoskeletal model, Software for Interactive Musculoskeletal Modeling, SIMM (Musculographics Corp.), the joint angle data was obtained. The reaction force data was filtered through a fourth-order Butterworth low-pass filter with a cut-off frequency of 20 Hz.

### Evaluation of Effect of Sensory Input on Muscle Synergy

In this study, the effects of visual and vestibular inputs on the spatiotemporal structure of muscle synergy were assessed. First, the spatial patterns of muscle synergy were studied to examine the utilization of similar muscle coordination. To this end, the similarities in the spatial patterns were calculated under different conditions. To begin, an average spatial pattern was obtained for each synergy in the individual conditions for each subject. Subsequently, these average spatial patterns were compared between two conditions to determine the similarity. Because spatial patterns were represented as vectors, the similarity was calculated as the correlation coefficient of the spatial pattern vectors **w**^*i*^ and **w**^*j*^, as expressed in the following equation:

$$s\left(w^{i},\;w^{j}\right)=\frac{\sum_{k=1}^{n}\left(w_{k}^{i}-{\bar{w}}^{i}\right)\left(w_{k}^{j}-{\bar{w}}^{j}\right)}{\sqrt{\left(\sum_{k=1}^{n}{\left(w_{k}^{i}-{\bar{w}}^{i}\right)}^{2})({\sum_{k=1}^{n}\left(w_{k}^{j}-{\bar{w}}^{j}\right)}^{2}\right)}},$$

where *i* and *j* represents a muscle synergy number obtained from either control, visual disturbance, or vestibular disturbance condition. The similarities of the spatial patterns were calculated using the same synergy obtained from different conditions. When the value was above 0.4, the spatial patterns should be similar (Ivanenko et al., 2005). When the similarity was obtained from all the subjects, then mean and standard deviation were calculated, and a 95% confidence interval was obtained to compare to the previous similarity threshold (0.4).

**Figure 2:**
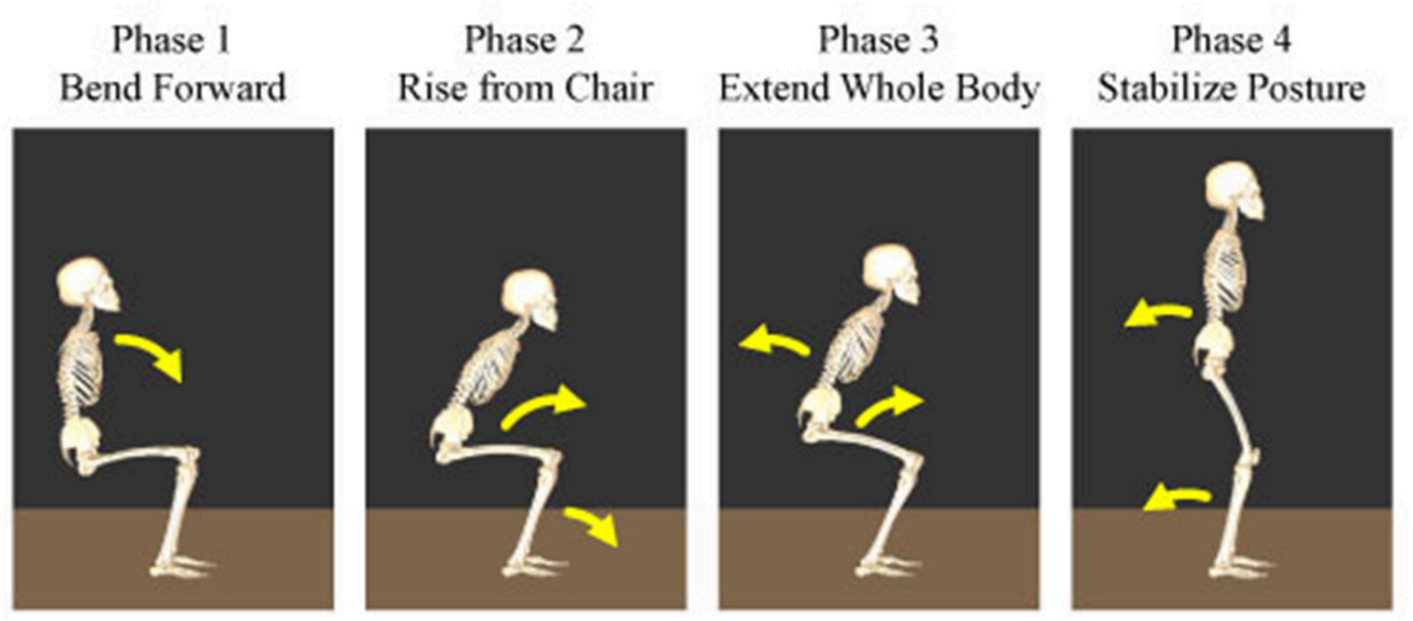
Four phases of the human sit-to-stand motion. In phase 1, humans flex their upper body to generate momentum and initiate the sit-to-stand motion. In phase 2, they raise their hip from the chair and transfer momentum. In phase 3, humans extend their whole body upward. In phase 4, they move their body backward to stabilize their posture.

To evaluate the differences in the temporal structures, their amplitude and duration were compared under different conditions. Particularly, the human sit-to-stand motion was divided into four phases and the effects of the visual and vestibular inputs on the muscle synergy were investigated based on these phases. The following are the four phases reported in the previous study: In phase 1, the upper body is flexed to generate momentum and initiate the sit-to-stand motion. In phase 2, they raise their hips from a chair and transfer momentum. In phase 3, they extend their entire body upward. In phase 4, they move their body backward to stabilize the posture. The starting times of the four phases of the sit-to-stand motion are depicted in Figure 2.

In this study, the body trajectory and reaction force data were used to identify the starting time *T*_*i* {*i* = 1⋯4}_ and duration *D*_*i* {*i* = 1⋯4}_ of each phase. Phase 1 is the beginning of the movement and its start time was decided from the marker position on the shoulder. The start of phase 1, *T*_1_, was determined by when the acceleration of the shoulder marker in the former direction exceeded a defined threshold. Phase 2 was defined as the hip rise from a chair, and thus, its start time *T*_2_ was calculated as the time when the hip reaction force became 0 N. In phase 3, the body leans forward the most and begins moving upward. Thus, the time of minimum dorsiflexion of the ankle joint was defined as the start of phase 3, *T*_3_. Finally, phase 4 was defined as the period of time for posture-stabilization after standing-up. Therefore, the time at which the maximum vertical position of the shoulder marker was achieved was set as the starting time of phase 4, *T*_4_. The end of phase 4 cannot be determined explicitly, and thus, it was determined from the period of the former phases, phases 1 to 3. Specifically, the duration of phase 4, *D*_4_, was calculated using the following equation:

$$\begin{array}{l} D_{4}=\alpha \left(T_{4}-T_{1}\right), \end{array}$$

where α is the ratio of the phase 4 duration to the period of phases 1–3. In this study α was set to 0.2.

The starting time and duration of the four phases are denoted as *T*_*i* {*i* = 1, 2, 3, 4}_ and *D*_*i* {*i* = 1, 2, 3, 4}_, and the average amplitude $V_{i}^{j}$ of the synergy *j* in the phase *i* was obtained using the following equation:

$$\begin{array}{l} V_{i}^{j}=\frac{\overset{T_{i+1}}{\underset{T_{i}}{\sum }}h_{j}\left(t\right)}{D_{i}}. \end{array}$$

The average amplitude was obtained for all the synergies under the three sensory conditions. To calculate changes of the duration time of the phases, the average duration time of each phase was obtained under the control condition, denoted ${\hat{D}}_{i}$. Subsequently, changes of the durations of the phases in the visual and vestibular-disturbance conditions were obtained as the ratio of the duration in the sensory-disturbance condition to the time in the control condition as follows:

$$\begin{array}{l} {\Delta D}_{i}=\frac{D_{i}}{{\hat{D}}_{i}}. \end{array}$$

Note that in the above equation, the numerator, *D*_*i*_, indicates the duration of phase *i* in the visual- and vestibular-disturbance conditions, and the denominator, ${\hat{D}}_{i}$, indicates the duration obtained in the control condition.

Both the evaluation parameters were used to examine whether the durations or amplitudes of the synergies changed due to the disturbed sensory input. One factor analysis of variance, ANOVA, was used to assess the effects of different sensory conditions on the amplitude and duration of the temporal patterns of each muscle synergy. If there was statistically significant difference (*p* < 0.05), then a *post hoc* test (Tukey's test) was employed. Analysis was performed on both sides of the subjects. To clarify the effect of the visual input, muscle synergies between the control and visual-disturbance conditions were compared. Similarly, the visual-disturbance and vestibular-disturbance conditions were compared to evaluate the effect of vestibular input.

### Subjects and Ethical Statement

Our experiments were performed by seven healthy subjects (six males 20–30 years old and one female 30–40 years old). This study was carried out in accordance with the recommendations of the guidelines for studies on humans, Environmental Health and Safety Office, School of Engineering, the University of Tokyo. The protocol was approved by the Institute Review Board (IRB) of the University of Tokyo. All subjects gave written informed consent in accordance with the Declaration of Helsinki.

## Results

**Figure 3:**
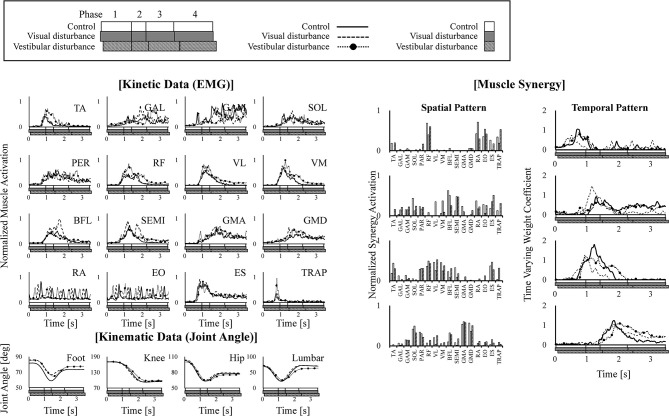
Example of kinematic (joint angle) and kinetic (EMG) data are shown in the left of the figure. The extracted four muscle synergies are shown in the right of the figure. The joint angles are defined as the angle between the link and the horizontal axis. The data obtained under control, visual-disturbance, and vestibular-disturbance conditions are shown as solid, dashed, and dotted lines with circle markers, respectively. In each graph, white, gray, and black squares with diagonal lines below the horizontal axis, respectively show duration time of four phases.

Typical kinematic (joint angle) and kinetic (EMG) data and the corresponding spatiotemporal pattern extracted from the subjects are shown in Figure 3. The figure also shows that these four modules activated sequentially from muscle synergy 1–4. Changes in these spatiotemporal patterns due to the disturbed sensory input were investigated.

**Figure 4:**
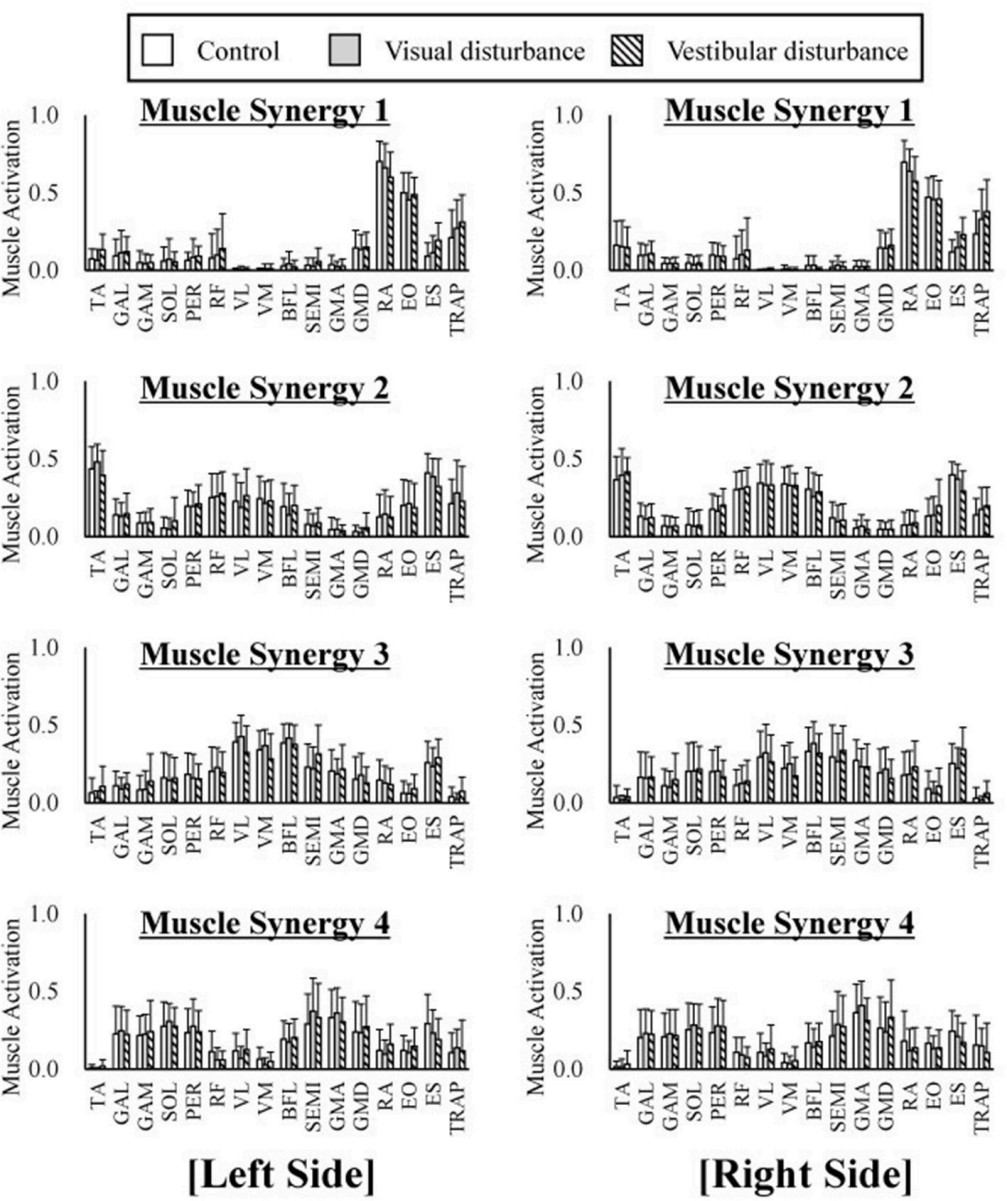
The average and standard deviation of the spatial patterns of the muscle synergies under three different condition are displayed. Four graphs in the left of the figure represent the spatial patterns extracted from the left side of the subjects whereas the right of the figure indicate the spatial patterns extracted from the right side. This graph is based on 120 samples in total. Each spatial pattern has specific contribution to the sit-to-stand motion. The muscle synergy 1 activates RA and EO to flex upper body. The muscle synergy 2 activates TA, VL, VM, and VM to rise hip from a chair and move forward. The muscle synergy 3 activates VL, VM, and ES to extend whole body. At last the muscle synergy 4 activates GAL, GAM, SOL, GMA, and GMD to stabilize posture. It has been shown that spatial patterns are similar regard less of different sensory condition.

The average and standard deviation of the spatial patterns of the muscle synergies are displayed in Figure 4 obtained under the control, visual-disturbance, and vestibular-disturbance conditions. The spatial patterns obtained from the left side muscles (the ipsilateral to the ear used for the caloric test) are shown in the left in Figure 4 and the spatial patterns obtained from the right side muscles (the contralateral to the ear used for the caloric test) are shown in the right side of Figure 4. Some characteristic activation was found in each synergy. In muscle synergy 1, muscles RA and EO were mostly activated to flex the lumbar joint. Muscle synergy 2 activated the TA to dorsiflex the ankle and activated the RF, VL, and VM to extend the knee joint. This synergy contributed to the rising of the hip from a chair and moving the body forward. In muscle synergy 3, the VL, VM, BFL, SEMI, GMA, GMD, and ES were activated to contribute to the extension of the knee, hip, and lumber joints to move upward. Muscle synergy 4 activated the GAL, GAM, SOL, PER, GMA, and GMD to plantarflex the ankle and extend the hip joint in the last phase of the sit-to-stand motion to decelerate the motion.

The similarities in the spatial patterns between the three conditions are presented in Table 1. These similarities for the control and visual-disturbance conditions are listed in Table 1A, and those for the visual-disturbance and vestibular-disturbance conditions are listed in Table 1B. These values are the average and standard deviation of the similarities in the spatial patterns in each subject. In addition to the average and standard deviation, a 95% confidence interval was also computed to evaluate the confidence level. In the previous study (Ivanenko et al., 2005), muscle synergies were considered to be similar when the coefficient of correlation between two muscle synergies was above 0.4. Our results showed that, regardless of the comparison between control and visual-disturbance conditions and between visual-disturbance and vestibular-disturbance conditions, the similarities in the spatial patterns were above 0.4, implying that the participants likely employed similar spatial patterns.

**Table 1:** Similarity of spatial patterns.

		**Synergy 1**	**Synergy 2**	**Synergy 3**	**Synergy 4**
**(A) COMPARISON OF CONTROL AND VISUAL-DISTURBANCE CONDITIONS**					
Right	Mean ±*SD*	0.92 ± 0.04	0.95 ± 0.04	0.92 ± 0.07	0.87 ± 0.13
	95% Upper CL	0.96	0.99	0.99	0.99
	95% Lower CL	0.88	0.84	0.80	0.91
Left	Mean ±*SD*	0.92 ± 0.05	0.91 ± 0.08	0.91 ± 0.12	0.94 ± 0.04
	95% Upper CL	0.97	0.99	1.00 (1.03)	0.98
	95% Lower CL	0.87	0.84	0.80	0.91
**(B) COMPARISON OF VISUAL-DISTURBANCE AND VESTIBULAR-DISTURBANCE CONDITIONS**					
Right	Mean ±*SD*	0.90 ± 0.07	0.83 ± 0.10	0.73 ± 0.23	0.75 ± 0.15
	95% Upper CL	0.97	0.92	0.95	0.88
	95% Lower CL	0.84	0.74	0.52	0.61
left	Mean ±*SD*	0.90 ± 0.06	0.82 ± 0.10	0.82 ± 0.18	0.81 ± 0.11
	95% Upper CL	0.95	0.91	0.98	0.91
	95% Lower CL	0.85	0.73	0.66	0.71

*The similarities between the control and visual-disturbance conditions are listed in (A), and those between the visual-disturbance and vestibular-disturbance conditions are listed in (B). The tables show means, standard deviations, and 95% upper and lower confidence intervals for each synergy*.

**Figure 5:**
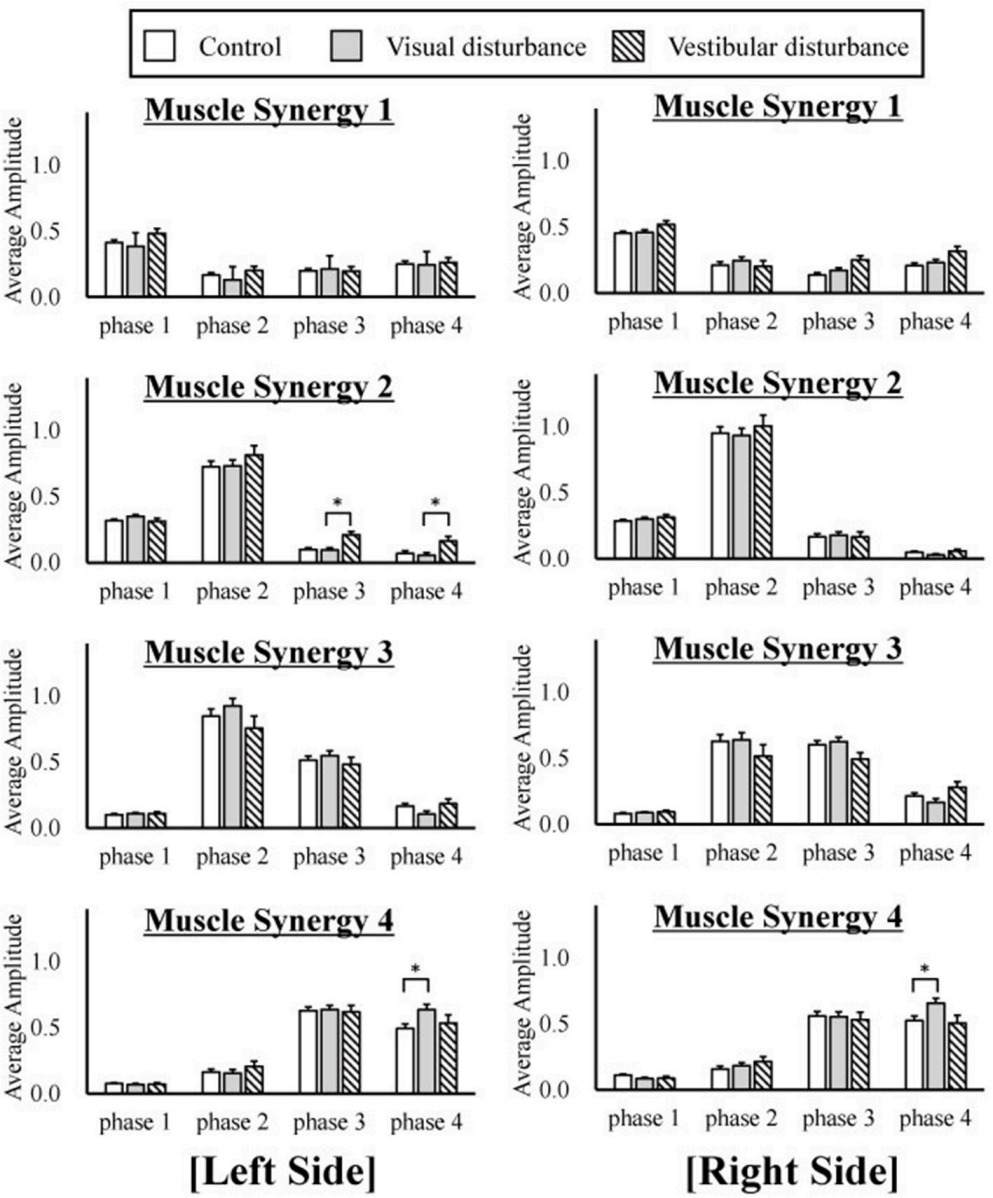
The average and standard deviation of the temporal patterns of the muscle synergies under three different conditions are displayed. The amplitudes of the temporal patterns were averaged in each phase of the sit-to-stand motion. The results from the left are displayed on the left of the figure, and the results from the right side are displayed in the right. This graph is based on 120 samples in total. A statistical test showed that muscle synergy 2 extracted from the left side had a larger amplitude during phases 2 and 3 under the vestibular-disturbance condition than under the visual-disturbance condition. In addition, the amplitude of the muscle synergy 4 increased under the visual-disturbance condition than under the control condition in both sides.

**Figure 6:**
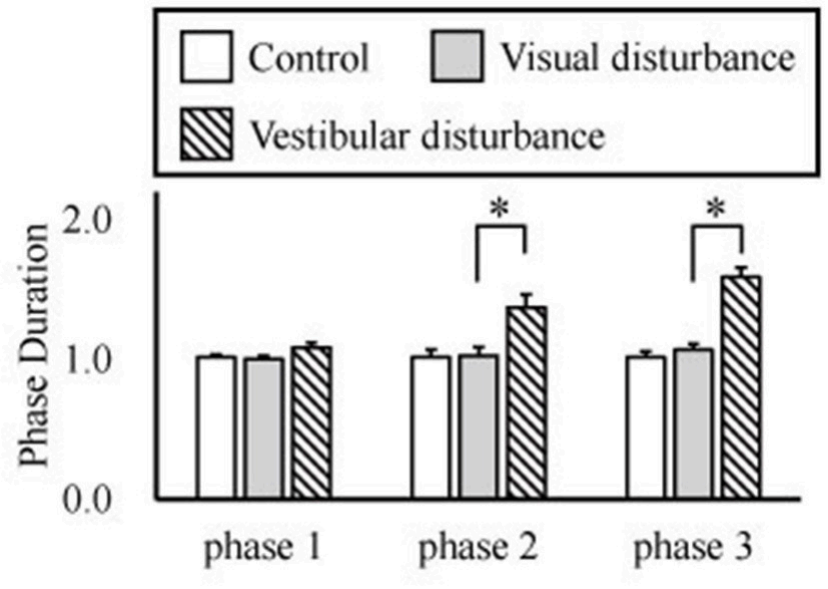
Average and standard deviation of the durations of the phases in sit-to-stand motion. The duration of phase 4 was determined by the duration of phases 1–3, and thus, it is not shown above. According to the results of the statistical test, the durations of phase 2 and 3 were significantly longer under the vestibular-disturbance condition than under the visual-disturbance condition.

The amplitudes of the temporal patterns on the right and left sides were averaged in each phase of the sit-to-stand motion, and the results are displayed in Figure 5. Detailed results of the statistical tests are shown in Table 2. These results suggest that muscle synergies contributed to each phase. Muscle synergies 1 and 2 were activated in phases 1 and 2, respectively. Muscle synergy 3 was mostly activated in phases 2 and 3, and muscle synergy 4 was activated in phases 3 and 4. Based on the ANOVA and *post-hoc* test, the activation of muscle synergy 4 was significantly higher in phase 4 under the visual-disturbance condition than under the control condition in both sides of the body. Activation of muscle synergy 2 was significantly higher in the left side in phases 3 and 4 under the vestibular-disturbance condition than under the visual-disturbance condition. The change in the durations of the phases were calculated for each condition, and the results are shown in Figure 6. Detailed results of the statistical tests are shown in Table 3. According to the statistical tests, the durations of phases 2 and 3 were significantly longer under the vestibular-disturbance condition than under the visual-disturbance condition.

Table 2Amplitudes of temporal patterns compared for each phase under the three conditions.

**Table:** 

**Phase**	**Synergy**		***p*-value**	***F*-statistics**		***p*-value**	***F*-statistics**
**(A) STATISTIC VALUES OF ANOVA FOR EACH SYNERGY IN EACH PHASE UNDER THE THREE CONDITIONS**							
1	1	Right	0.128	2.091	Left	0.095	2.400
	2	Right	0.406	0.908	Left	0.120	2.158
	3	Right	0.549	0.603	Left	0.791	0.235
	4	Right	0.091	2.450	Left	0.745	0.296
2	1	Right	0.584	0.541	Left	0.175	1.770
	2	Right	0.779	0.251	Left	0.546	0.608
	3	Right	0.458	0.787	Left	0.286	1.267
	4	Right	0.391	0.946	Left	0.538	0.623
3	1	Right	0.004^*^	5.708	Left	0.856	0.155
	2	Right	0.933	0.069	Left	0.000^*^	8.899
	3	Right	0.077	2.618	Left	0.541	0.618
	4	Right	0.916	0.088	Left	0.954	0.047
4	1	Right	0.054	2.988	Left	0.897	0.109
	2	Right	0.101	2.340	Left	0.017^*^	4.205
	3	Right	0.080	2.587	Left	0.083	2.544
	4	Right	0.015^*^	4.390	Left	0.034^*^	3.467

**Table:** 

**Phase**	**Synergy**		**Group 1**	**Group 2**	***p*****-value**
**(B) STATISTIC VALUES OF** ***Post-hoc*** **TESTS BETWEEN CONTROL AND VISUAL-DISTURBANCE CONDITIONS AND VISUAL-DISTURBANCE AND VESTIBULAR-DISTURBANCE CONDITIONS**					
3	1	Right	Control	Visual-disturbance	0.358
			Visual-disturbance	Vestibular-disturbance	0.062
3	2	Left	Control	Visual-disturbance	0.995
			Visual-disturbance	Vestibular-disturbance	0.000^*^
4	2	Left	Control	Visual-disturbance	0.851
			Visual-disturbance	Vestibular-disturbance	0.015^*^
4	4	Right	Control	Visual-disturbance	0.022^*^
			Visual-disturbance	Vestibular-disturbance	0.076
		Left	Control	Visual-disturbance	0.027^*^
			Visual-disturbance	Vestibular-disturbance	0.379

Statistic values of ANOVA are shown in (A) and p-values of post hoc test (Tukey's test) are shown in (B). An asterisk

* mark is added next to the p-value when there is a statistical significance (p < 0.05)

Table 3Duration of temporal patterns compared for each phase under the three conditions.

**Table:** 

	***p*-value**	***F*-statistics**
**(A) STATISTIC VALUES OF ANOVA FOR EACH PHASE UNDER THE THREE CONDITIONS**		
Phase 1	0.110	2.250
Phase 2	0.004^*^	5.823
Phase 3	0.000^*^	30.003

**Table:** 

	**Group 1**	**Group 2**	***p*****-value**
**(B) STATISTIC VALUES OF THE** ***Post-hoc*** **TEST BETWEEN CONTROL AND VISUAL-DISTURBANCE CONDITIONS AND VISUAL-DISTURBANCE AND VESTIBULAR-DISTURBANCE CONDITIONS**			
Phase 2	Control	Visual-disturbance	0.989
	Visual-disturbance	Vestibular-disturbance	0.007^*^
Phase 3	Control	Visual-disturbance	0.636
	Visual-disturbance	Vestibular-disturbance	0.000^*^

Statistic values of ANOVA are shown in (A) and p-values of post hoc test (Tukey's test) are shown in (B). An asterisk

* *mark is added next to the p-value when there is a statistical significance (p < 0.05)*.

## Discussion

Our analysis of the spatial patterns of muscle synergies showed that the same spatial patterns may be utilized despite disturbed visual and vestibular inputs. However, the temporal structures of the muscle synergies were affected by the disturbed sensory input. When the visual input was disturbed, the amplitude of muscle synergy 4 increased in phase 4, whereas other muscle synergies did not differ significantly in any phase. Under the vestibular-disturbance condition, it was found that duration of phases 2 and 3 increased, resulting in longer activation of muscle synergies 2 and 3, which mostly activated during these two phases. Moreover, it was found that amplitude of muscle synergy 2 was larger in this condition.

Similar to our previous study (Yang et al., 2017), in which different strategies of the sit-to-stand motion were investigated, and to another study (Ivanenko et al., 2005), in which human locomotion was analyzed, the spatial patterns of muscle synergies were found to be similar. It was also reported recently (Takei et al., 2017; Desrochers et al., 2018) that this muscle coordination is coded in the spinal cord and is unchanged. Our current result also supports the findings that similar spatial patterns coded in the spinal cord can be utilized to achieve adaptive movements despite disturbed sensory input. Comparisons between the control and visual-disturbance conditions suggested that visual information is not utilized to correct muscle synergies between the beginning of the motion and body extension (phases 1–3). However, additional activation was required in the last phase, in which the body posture must be stabilized. This suggests the possibility that muscle synergies 1–3 were not only affected by visual input but that muscle synergy 4 required greater activation to compensate for the disturbed visual input. However, vestibular input primarily changed the duration of muscle synergies 2 and 3. Muscle synergies 2 and 3 contributed to the movement of the hip rising from the chair and that of whole body extension. In both movements, the vertical upward direction is needed to be recognized correctly to localize the body posture. Therefore, the sense of verticality was impaired under the vestibular-disturbance condition, and thus, without vestibular input, the participants could not raise their hips and extend their bodies as fast as under the other conditions. In other words, the duration of muscle synergies 2 and 3 was prolonged to balance in unstable conditions. This result is consistent with the results implying that neural system prolonged duration of muscle synergies to adopt a different motion strategy in locomotion under unstable conditions (Martino et al., 2015). Furthermore, it was found that the amplitude of the muscle synergy 2 increased in phases 3 and 4. This implied that vestibular input contributes to the determination of the completion time of muscle synergy 2 as well as the amplitude. Thus, impaired vestibular input results in a larger amplitude of muscle synergy 2, even in phases 3 and 4. As reported in the previous study (Fitzpatrick et al., 1999), humans cannot recognize the direction of travel under the disturbed vestibular input and could not walk straight. In our experiment, subjects could complete the sit-to-stand motion, but it required more effort to control muscle synergies due to the lacking sense of verticality. This result suggests that the vestibular sensation contributes to ensuring the movement direction during phases 2 and 3, and the duration is changed without vestibular sensation.

In this study, visual input was disturbed by Frenzel glasses and a dark room. We utilized caloric tests to disturb the vestibular input. Similar to previous reports, the subjects could utilize other sensory inputs, including the somatosensory system and proprioception, to change the weight of other sensory input (sensory reweighting) (Maurer et al., 2000; Chiba et al., 2013). In our experiment, this sensory reweighting was believed to occur. In other words, humans may rely on other sensations under visual and vestibular-disturbance conditions. Our future study will investigate how the weight of each sensory input is changed when sensory inputs are disturbed.

## Conclusions

Human sit-to-stand motion was analyzed under different sensory input conditions, and the effects of the visual and vestibular sensory inputs on muscle synergies were studied. It was found that four similar spatial patterns of muscle coordination are utilized despite the disturbed visual and vestibular inputs. In contrast, it was revealed that the temporal structure of the muscle synergies varied with the disturbed sensory input. Our results suggest that humans do not depend much on visual input during the trunk flexion, hip raising from the chair, and entire body extension phases. However, posture stabilization required greater synergy activation under the visual-disturbance condition after the subjects completed the body extension phase. In contrast, vestibular input contributed to the sense of verticality, and the subjects utilized vestibular input to correct their posture while raising their hips from a chair and extending their bodies. One future study will examine the effects of different sensory inputs, such as the somatosensory input, proprioception, and pressure sensation of the hips and feet. Investigation of a wide range of sensory inputs would improve the understanding of the mechanisms of sit-to-stand motion.

## Data Availability Statement

The datasets for this manuscript are not publicly available because those contain private data. Requests to access the datasets should be directed to KY.

## Author Contributions

KY, QA, HY, YT, AtY, and HA contributed to the conception and design of this study. KY, QA, and ArY designed and performed the experiments. KY and QA analyzed the results. KY, QA, ArY, RC, and KT contributed to discussions of the results. KY and QA wrote sections of the manuscript. All authors contributed to the manuscript revision, and read and approved the submitted version.

### Conflict of Interest Statement

The authors declare that the research was conducted in the absence of any commercial or financial relationships that could be construed as a potential conflict of interest.
